# NMDA Receptors in Accumbal D1 Neurons Influence Chronic Sugar Consumption and Relapse

**DOI:** 10.1523/ENEURO.0029-21.2021

**Published:** 2021-05-14

**Authors:** Shoupeng Wei, Sarah Hertle, Rainer Spanagel, Ainhoa Bilbao

**Affiliations:** 1 Behavioral Genetics Research Group, Institute of Psychopharmacology, Central Institute of Mental Health, Heidelberg University, Mannheim, 68159, Germany; 2 Institute of Psychopharmacology, Central Institute of Mental Health, Heidelberg University, Mannheim, 68159, Germany

**Keywords:** addictive phenotypes, glutamate receptors, mesolimbic system, mice, sugar, transgenic models

## Abstract

Glutamatergic input via NMDA and AMPA receptors within the mesolimbic dopamine (DA) pathway plays a critical role in the development of addictive behavior and relapse toward drugs of abuse. Although well-established for drugs of abuse, it is not clear whether glutamate receptors within the mesolimbic system are involved in mediating chronic consumption and relapse following abstinence from a non-drug reward. Here, we evaluated the contribution of mesolimbic glutamate receptors in mediating chronic sugar consumption and the sugar-deprivation effect (SDE), which is used as a measure of relapse-like behavior following abstinence. We studied four inducible mutant mouse lines lacking the GluA1 or GluN1 subunit in either DA transporter (DAT) or D1R-expressing neurons in an automated monitoring system for free-choice sugar drinking in the home cage. Mice lacking either GluA1 or GluN1 in D1R-expressing neurons (*GluA1^D1CreERT2^* or *GluN1^D1CreERT2^*mice) have altered sugar consumption in both sexes, whereas *GluA1^DATCreERT2^* and *GluN1^DATCreERT2^*do not differ from their respective littermate controls. In terms of relapse-like behavior, female *GluN1^D1CreERT2^*mice show a more pronounced SDE. Given that glutamate receptors within the mesolimbic system play a critical role in mediating relapse behavior of alcohol and other drugs of abuse, it is surprising that these receptors do not mediate the SDE, or in the case of female *GluN1^D1CreERT2^* mice, show an opposing effect. We conclude that a relapse-like phenotype of sugar consumption differs from that of drugs of abuse on the molecular level, at least with respect to the contribution of mesolimbic glutamate receptors.

## Significance Statement

Here, we provide evidence from various inducible and site-specific transgenic mouse models that glutamate receptors within the mesolimbic dopamine (DA) system do not play a critical role in relapse behavior in chronically sugar-drinking male and female mice. This differentiates a natural reward from drugs of abuse on the molecular level, as mesolimbic NMDA and AMPA receptors are essential for drug-induced neuroplasticity and subsequent relapse behavior.

## Introduction

It is assumed that the problematic chronic use of sugar, similar to chronic consumption of drugs of abuse, can lead to an addictive-like phenotype. However, the concept of “sugar addiction” is controversial and only a few studies have attempted to determine the addictive properties of sugar using rigorous scientific criteria (Avena et al., 2009; Wiss et al., 2018).

These studies suggest that behavioral phenotypes associated with chronic consumption of drugs of abuse and sugar consumption are similar with respect to withdrawal responses, compulsive over-consumption, craving, and loss of control (Avena et al., 2009; Wiss et al., 2018). After deprivation even relapse behavior can ensue. Thus, rats trained for 28 d to drink a sucrose solution and deprived for 14 d displayed a sugar-deprivation effect (SDE; Avena et al., 2005). In a more recent study (Wei et al., 2021), the addictive-like properties of sugar were systematically examined in male and female mice using established paradigms and models from the drug addiction field (Sanchis-Segura and Spanagel, 2006; Wei et al., 2021). In this study, female mice were more vulnerable to the addictive-like properties of sugar than male mice, showing higher long-term, excessive sugar drinking, and a more pronounced relapse-like sugar consumption as assessed by measuring the SDE (Wei et al., 2021). The deprivation effect is a measure of consumption during a relapse-like situation in the addiction field (Vengeliene et al., 2014; Spanagel, 2017).

Given the similarities of phenotypes for the chronic use of drugs of abuse and sugar, we speculated that there may also be similarities on the molecular level. In the addiction field, there is strong evidence that an interaction between the glutamatergic and mesolimbic dopamine (DA) systems is critical for mediating the reinforcing effects of drugs of abuse and consequently addictive behavior and relapse (Gass and Olive, 2008). In particular, glutamatergic synapses on DA neurons in the ventral tegmental area (VTA) and D1 receptor-expressing medium spiny neurons (MSNs) of the nucleus accumbens (NAc) both modulate the reinforcing properties of drugs of abuse and reward-dependent learning processes (Lüscher and Malenka, 2011; Lüscher, 2013; Scofield et al., 2016). In support of this, disruption of NMDA receptors in midbrain DA neurons abolishes enduring cocaine-induced plasticity in the NAc, thus reducing the incubation of craving and subsequent relapse behavior (Engblom et al., 2008; Mameli et al., 2009). Furthermore, using different mutant mouse lines that lack GluN1 and GluA1 receptor subunits in DA transporter (DAT) and D1R-expressing neurons, respectively, it was shown that GluN1 and GluA1 receptor subunits within these neuronal subpopulations mediate the alcohol-deprivation effect (ADE), which is a measure for relapse behavior (Eisenhardt et al., 2015a).

Some drug-induced neuroplastic changes within the mesolimbic system may also occur following consumption of natural rewards. For example, sucrose intake increases the phosphorylation and trafficking of accumbal AMPA receptor GluA1 subunits (Tukey et al., 2013) and alters the morphology of the MSNs (Klenowski et al., 2016). In addition, other studies have shown that a natural reward experience activates VTA DA cells and alters AMPA and NMDA receptor distribution and function in the NAc similar to psychostimulants (Pitchers et al., 2012; Beloate et al., 2016). Therefore, mesolimbic glutamate receptors may, at least in part, be involved in mediating chronic sugar consumption and relapse following abstinence. Furthermore, there may be sex-dependent effects in sugar consumption and relapse, as female rats have increased levels of the AMPA receptor GluA1 and NMDA receptor NR1 subunits within the mesolimbic system after cocaine, methamphetamine or ethanol self-administration, relative to male rats (Devaud and Alele, 2004; Bechard et al., 2018; Pena-Bravo et al., 2019).

The aim of the present study was to systematically examine the involvement of AMPA and NMDA receptors within the mesolimbic system in mediating chronic long-term sugar consumption and the SDE in a sex-dependent manner. Here, we generated inducible mutant mice expressing GluN1 or GluA1 mutations under the control of the DAT (*Slc6a3*) or D1 (*Drd1a*) promoter following the previously described procedure (Mameli et al., 2009; Parkitna et al., 2009, 2010; Eisenhardt et al., 2015a). We focused on AMPA and NMDA receptors in D1-receptor-containing MSNs, as several studies (Hikida et al., 2010; Lobo and Nestler, 2011; Calipari et al., 2016; Soares-Cunha et al., 2016; Ma et al., 2018; Bilbao et al., 2020) suggest that this neuronal population is more involved in mediating the chronic effects of drug of abuse and natural rewards than D2-containing MSNs. Using a fully automated, highly precise home cage monitoring system (Eisenhardt et al., 2015b) for sugar drinking in mice, we systematically examined *GluN1^DATCreERT2^*, *GluA1^DATCreERT2^*, *GluN1^D1CreERT2^*, and *GluA1^D1CreERT2^* male and female mice in a long-term free-choice sugar drinking procedure and studied the SDE following an abstinence phase.

## Materials and Methods

### Animals

We generated mutant mice expressing GluN1 or GluA1 mutations under control of the DAT (*Slc6a3*) or D1 (*Drd1a*) promoter following the previously described procedure (Mameli et al., 2009; Parkitna et al., 2009, 2010; Eisenhardt et al., 2015a). In short, *GluN1^DATCreERT2^*, *GluA1^DATCreERT2^*, *GluN1^D1CreERT2^*, and *GluA1^D1CreERT2^* mice were generated by crossing mice with an inducible Cre-recombinase under the DAT- or D1-promoter with mice carrying floxed alleles for GluN1or GluA1. The *DATCreERT2* and *D1CreERT2* mice were generated by recombining a construct containing an improved Cre-recombinase fused to a modified ligand binding domain of the estrogen receptor (CreERT2) into a bacterial artificial chromosome containing the gene encoding DAT (*Slc6a3*) or D1 (*Drd1a*) by recombineering. *GluN1^fl/fl^* and *GluA1^fl/fl^* mice, having exons 11–18 of the *Grin1* or exon 11 of the *Gria1* alleles, respectively, flanked with loxP sites were generated by gene targeting in embryonic stem cells (Zamanillo et al., 1999; Niewoehner et al., 2007). For induction of the mutation, mice were treated with 1 mg of tamoxifen dissolved in neutral oil intraperitoneally twice a day for five consecutive days (Erdmann et al., 2007). Mice were treated with tamoxifen at an age of 8–10 weeks old and were allowed to recuperate for at least three weeks before experiments started. For genotyping of the DATCreERT2 and D1CreERT2 transgene, we used the primers GGC TGG TGT GTC CAT CCC TGA A and GGT CAA ATC CAC AAA GCC TGG CA. The GluN1 and GluA1 flox variants were genotyped using the primers GGA CAG CCC CTG GAA GCA AAA T and GGA CCA GGA CTT GCA GTC CAA AT for GluN1, and CAC TCA CAG CAA TGA AGC AGG AC and CTG CCT GGG TAA AGT GAC TTG G for GluA1. For all experiments, adult male and female *GluN1^DATCreERT2^*, *GluA1^DATCreERT2^*, *GluN1^D1CreERT2^*, and *GluA1^D1CreERT2^* and their wild-type littermate mice from at least six consecutive backcrosses with C57BL/6N were used (8–10 weeks at the beginning of the experiments). As controls, floxed littermates not carrying the Cre-recombinase were used.

Mice were single-housed in standard hanging cages at 21 ± 1°C and 50 ± 5% relative humidity on a reversed 12/12 h light/dark cycle, with lights on at 7:30 P.M. The animals were provided with standard rodent food (Altromin Spezialfutter GmbH & Co, LASQC diet Rod16-H. Composition: cereals, vegetable by-products, minerals, oils and fats, yeast; crude nutrients: 16.30% crude protein, 4.30% crude fat, 4.30% crude fiber, 7.00% crude ash), a bottle containing 5% (w/v) sugar solution during the long-term sugar paradigms (see below for details) and tap water *ad libitum*. All the experiments were performed in the dark cycle. All mice were handled on a daily basis before starting the experiments and were habituated to the behavioral testing environments. Procedures for this study complied with the regulations covering animal experimentation within the European Union (European Communities Council Directive 86/609/EEC) and Germany (Deutsches Tierschutzgesetz) and the experiment was approved by the German animal welfare authorities (Regierungspräsidium Karlsruhe).

### Home cage two-bottle free-choice sugar drinking and assessment of relapse-like drinking by means of the SDE

For this experiment 360 mice were used in total, 49 *GluN1^DATCreERT2^*(25 males and 24 females), 41 *GluN1^D1CreERT2^*(21 males and 20 females), 53 *GluA1^DATCreERT2^*(26 males and 27 females), 37 *GluA1^D1CreERT2^*(17 males and 20 females), and 180 respective control littermates (90 males and 90 females) were used. Mice had continuous free-choice access to a bottle containing a sugar solution (sucrose 5% w/v) and a bottle with tap water in the homecage for eight weeks. During the last 3 d of sugar exposure, sugar and water intake and locomotion were recorded using a drinkometer system (Eisenhardt et al., 2015a,b; Bilbao et al., 2019) and were used as baseline for comparison with the SDE. Mice were afterward deprived from sugar for 12–15 d, during which they only had access to two bottles of tap water. After the deprivation period, the SDE was tested for 24 h by reintroducing the sugar bottle.

### Assessment of drinking patterns by a fully automated drinkometer device

Sugar and water intake, preference over water and locomotor activity were measured during baseline and SDE measurements with a fully automated, highly precise monitoring system as described previously (INFRA-E-MOTION; Eisenhardt et al., 2015a,b; Bilbao et al., 2019). Briefly, during recording, the standard lid of the mouse home cage was replaced with the drinkometer lid containing two holes for special drinkometer bottles with a curved bottleneck and different tips for water (0.8 mm opening) and sugar (1.5 mm opening) solutions. The drinkometer system was configured to sample every 4 min, the amount (g) of sugar and water each mouse consumed. Sugar and water intake, preference over water and locomotor activity were calculated every 4 h to assess circadian drinking patterns and to obtain a temporal dissection of the SDE. The SDE in mice is usually short-lasting (Vengeliene et al., 2014) and therefore the first 4 h during the SDE provide the most valid measurement (Eisenhardt et al., 2015a).

Sugar (g/kg) and water (ml) intake, sugar preference (% of total fluid intake) and locomotor activity were calculated per day. During baseline and SDE measurements, sugar and water intake and locomotion were additionally calculated in 4-h time intervals. Baselines were calculated as the mean of the last 3 d of baseline recording.

### Statistics

Statistical analyses were performed by one-way or two-way ANOVA with repeated measures and Newman–Keuls test for *post hoc* comparisons using Statistica 10 (StatSoft). All values are given as mean ± SEM, and statistical significance was set at *p *<* *0.05.

The ANOVA model for the long-term free-choice home cage drinking and SDE contained the fixed effects of sugar deprivation (baseline and relapse), gene (wild-type and GluA1 or GluN1), and the interaction deprivation × gene.

## Results

### Specific GluN1 receptor subunit gene inactivation

**Figure 1.**
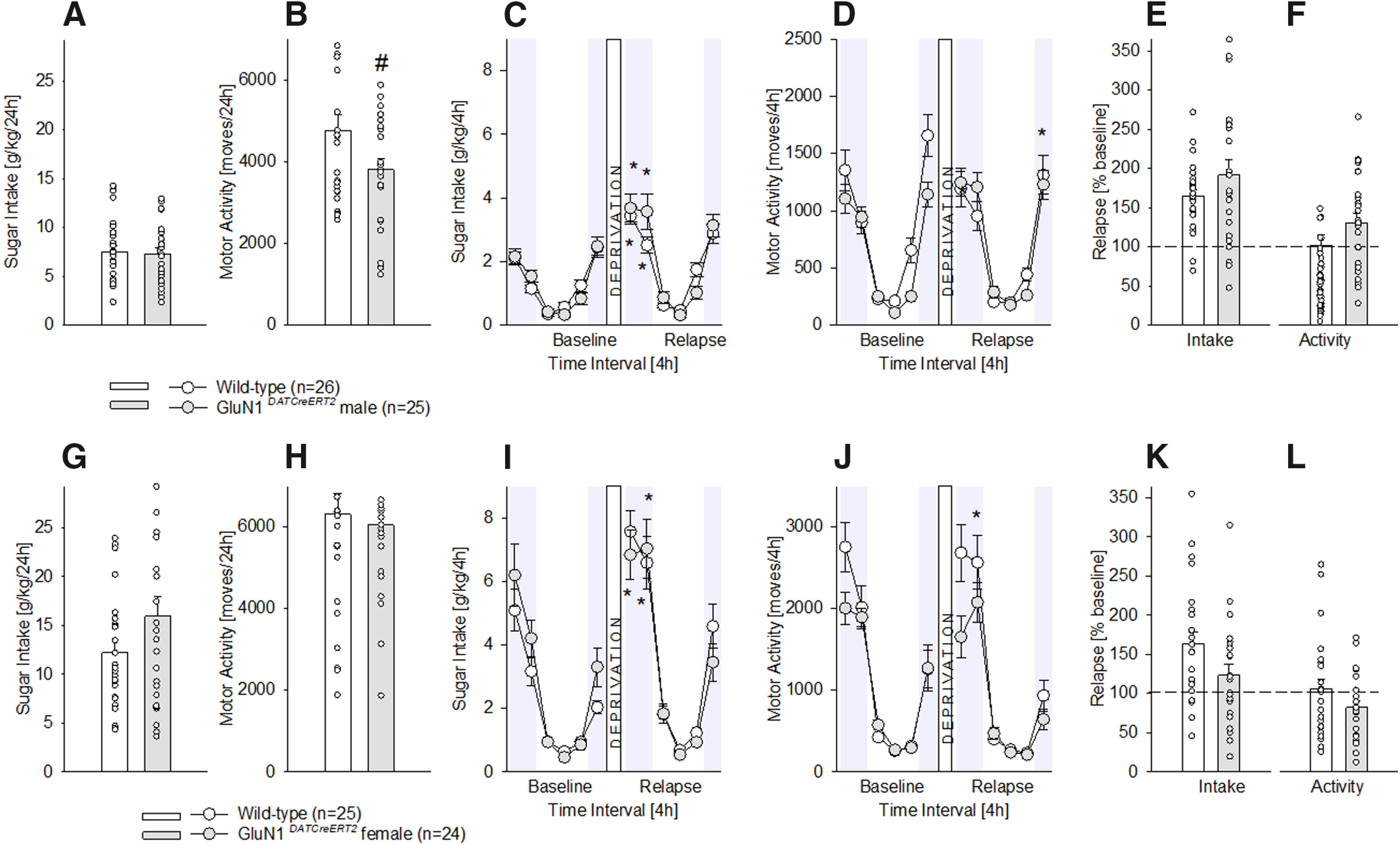
Characterization of chronic sugar drinking, SDE, and locomotor activity in male and female *GluN1^DATCreERT2^* mice. ***A***, ***G***, Baseline sugar intake in g/kg/d after free-choice access to a bottle containing a sugar solution (5% w/v) and a bottle with tap water in the homecage for eight weeks in wild-type male (*n* = 26), female (*n* = 25), and *GluN1^DATCreERT2^* male (*n* = 25) and female (*n* = 24) mice. ***B***, ***H***, Baseline locomotor activity after eight weeks of free-choice sugar drinking. ***C***, ***I***, Diurnal drinking pattern during baseline sucrose consumption and during the SDE. ***D***, ***J***, Diurnal activity pattern during baseline sucrose consumption and during the SDE. ***E***, ***K***, Relapse behavior measured in % change from baseline during the first 4 h of the SDE. ***F***, ***L***, Relative change in locomotor activity during the first 4 h of the SDE in relation to baseline activity; * indicates significant changes (*p* < 0.001) in sucrose consumption or activity during the SDE when compared with baseline; # indicates significant (*p* < 0.05) genotype differences.

After eight weeks of chronic, 24-h free-choice sugar exposure, male and female *GluN1^DATCreERT2^*mice did no differ from their wild-type littermates in the daily sugar intake (one-way ANOVA for Fig. 1*A*: *F*_(1,49)_ = 0.06, *p *=* *0.8 and for Fig. 1*G*: *F*_(1,47)_ = 2.7, *p *=* *0.1). Male *GluN1^DATCreERT2^*mice displayed a decreased baseline locomotion compared with wild-types (*F*_(1,49)_ = 4.4, *p *<* *0.05; Fig. 1*B*), which was not the case for females, showing no difference between genotypes (*F*_(1,47)_ = 0.2, *p *=* *0.7; Fig. 1*H*).

A period of sugar deprivation significantly increased the sugar intake in all mutants, and the respective wild-type mice (Fig. 1*C*,*I*), indicative of a SDE (two-way ANOVA, deprivation effect for Fig. 1*C*: *F*_(11,539)_ = 62.3, *p *<* *0.0001 and for Fig. 1*I*: *F*_(11,517)_ = 72.8, *p *<* *0.0001). The dissection of the baseline and SDE drinking into 4-h time interval points showed the typical diurnal pattern of intake, characterized by higher drinking during the dark, active phase, and lower drinking during the light, inactive phase of the day. Specifically, during the SDE (i.e., relapse), sugar intake was strongly pronounced during the first 4–8 h of re-exposure and lasted not longer than 24 h in all genotypes (two-way ANOVA, gene effect for Fig. 1*C*: *F*_(1,49)_ = 1.3, *p *=* *0.7 and for Fig. 1*I*: *F*_(1,47)_ = 0.07, *p *=* *0.8, Neuman–Keuls *post hoc p* < 0.001). A similar diurnal pattern was observed in the locomotor activity (Fig. 1*D*,*J*) during baseline and SDE. A period of sugar deprivation also influenced locomotor activity (deprivation effect for Fig. 1*D*: *F*_(11,539)_ = 58.5, *p *<* *0.0001 and for Fig. 1*J*: *F*_(11,517)_ = 66.2, *p *<* *0.0001), by increasing locomotion in mutant males and in wild-type females at the end and the beginning of the SDE, respectively, a phenomenon not related to *GluN1* mutations (gene effect for Fig. 1*D*: *F*_(1,49)_ = 1.6, *p *=* *0.2 and for Fig. 1*J*: *F*_(1,47)_ = 2.3, *p *=* *0.1, Neuman–Keuls *post hoc p* < 0.001). Supporting these results, the percentage of relapse over baseline during the first 4 h of sugar re-exposure for intake and locomotion (Fig. 1*E*,*K*, *F*,*L*) indicated similar SDE magnitude in mutant and control mice (one-way ANOVA for Fig. 1*E*: *F*_(1,49)_ = 1.7, *p *=* *0.2 and for Fig. 1*K*: *F*_(1,47)_ = 3.8, *p *=* *0.06; for Fig. 1*F*: *F*_(1,49)_ = 2.5, *p *=* *0.1 and for Fig. 1*L*: *F*_(1,47)_ = 2.5, *p *=* *0.1).

**Figure 2.**
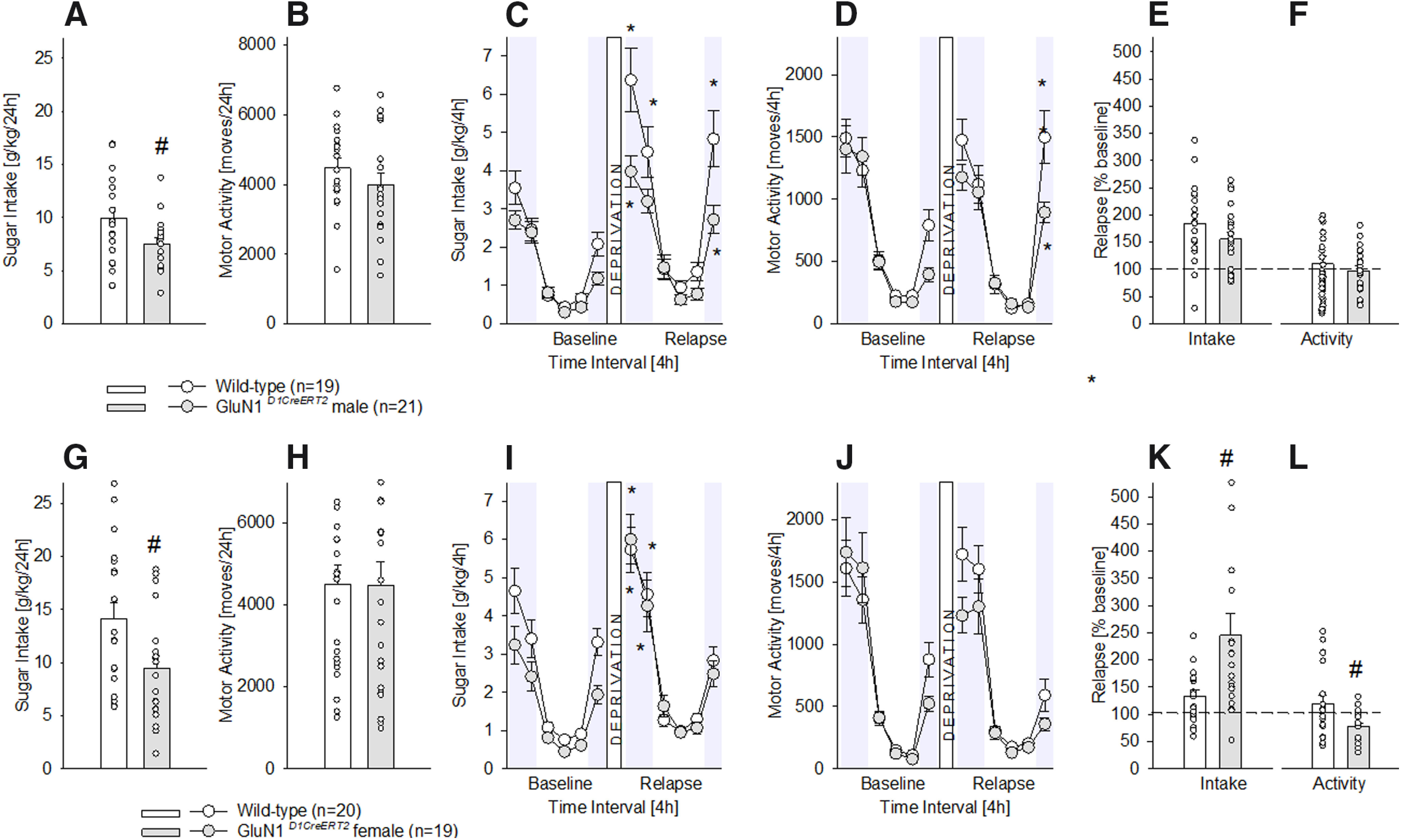
Characterization of chronic sugar drinking, SDE, and locomotor activity in male and female *GluN1^D1CreERT2^* mice. ***A***, ***G***, Baseline sugar intake in g/kg/d after free-choice access to a bottle containing a sugar solution (5% w/v) solution and a bottle with tap water in the homecage for eight weeks in wild-type male (*n* = 19), female (*n* = 20), and *GluN1^D1CreERT2^* male (*n* = 21) and female (*n* = 19) mice. ***B***, ***H***, Baseline locomotor activity after eight weeks of free-choice sugar drinking. ***C***, ***I***, Diurnal drinking pattern during baseline sucrose consumption and during the SDE. ***D***, ***J***, Diurnal activity pattern during baseline sucrose consumption and during the SDE. ***E***, ***K***, Relapse behavior measured in % change from baseline during the first 4 h of the SDE. ***F***, ***L***, Relative change in locomotor activity during the first 4 h of the SDE in relation to baseline activity; * indicates significant changes (*p* < 0.05) in sucrose consumption or activity during the SDE when compared with baseline; # indicates significant (*p* < 0.05 or *p* < 0.01 for ***A***, ***G***, ***L***, ***K***, respectively) genotype differences.

In contrast to the DAT-containing neurons, *GluN1* mutation onto D1-containing neurons (*GluN1^D1CreERT2^*mice) had an effect on chronic sugar drinking. As depicted in Figure 2, male (Fig. 2*A*) and female (Fig. 2*G*) *GluN1^D1CreERT2^*mice showed a significant decrease in the total, 24-h free-choice sugar drinking (one-way ANOVA for Fig. 2*A*: *F*_(1,38)_ = 4.5, *p *<* *0.05 and for Fig. 2*G*: *F*_(1,37)_ = 5.9, *p *<* *0.05). No differences were found between genotypes in locomotor activity at baseline (Fig. 2*B*: *F*_(1,38)_ = 1.3, *p *=* *0.3 and Fig. 2*H*: *F*_(1,37)_ = 0.001, *p *=* *1).

Furthermore (and in contrast to the DAT-containing neurons), *GluN1* mutation onto D1-containing neurons (*GluN1^D1CreERT2^* mice) had an impact on the SDE. Thus, although a period of sugar deprivation significantly increased the sugar intake in all mice (Fig. 2*C*,*I*), indicating a SDE (two way ANOVA, deprivation effect for Fig. 2*C*: *F*_(11,418)_ = 63.5, *p *<* *0.0001 and for Fig. 2*I*: *F*_(11,407)_ = 66.6, *p *<* *0.0001), statistical analysis also showed a gene × deprivation interaction effect (for Fig. 2*C*: *F*_(11,418)_ = 4.4, *p *<* *0.0001 and for Fig. 2*I*: *F*_(11,407)_ = 1.9, *p *<* *0.05, Neuman–Keuls *post hoc p* < 0.05). Again, the SDE was strongly pronounced during the first 4–8 h of re-exposure and lasted not longer than 24 h in all mice. Locomotor activity (Fig. 1*D*,*J*) was also influenced by the SDE, with males showing increased activity at the end of the SDE period (deprivation effect for Fig. 1*D*: *F*_(11,418)_ = 55.3, *p *<* *0.0001, Neuman–Keuls *post hoc p* < 0.05 and for Fig. 1*J*: *F*_(11,407)_ = 54.2, *p *<* *0.0001). When calculating the percentage of relapse over baseline during the first 4 h of sugar re-exposure for intake and locomotion, male *GluN1^D1CreERT2^*mice did not differ from their wild-type littermates (one-way ANOVA for Fig. 2*E*: *F*_(1,37)_ = 1.9, *p *=* *0.2 and for Fig. 2*F*: *F*_(1,37)_ = 0.3, *p *=* *0.6). However, in females, the SDE magnitude was higher for the intake (Fig. 2*K*: *F*_(1,37)_ = 8, *p *<* *0.01) and lower for the locomotion (Fig. 2*L*: *F*_(1,37)_ = 6.2, *p *<* *0.05).

### Specific GluA1 receptor subunit gene inactivation

**Figure 3.**
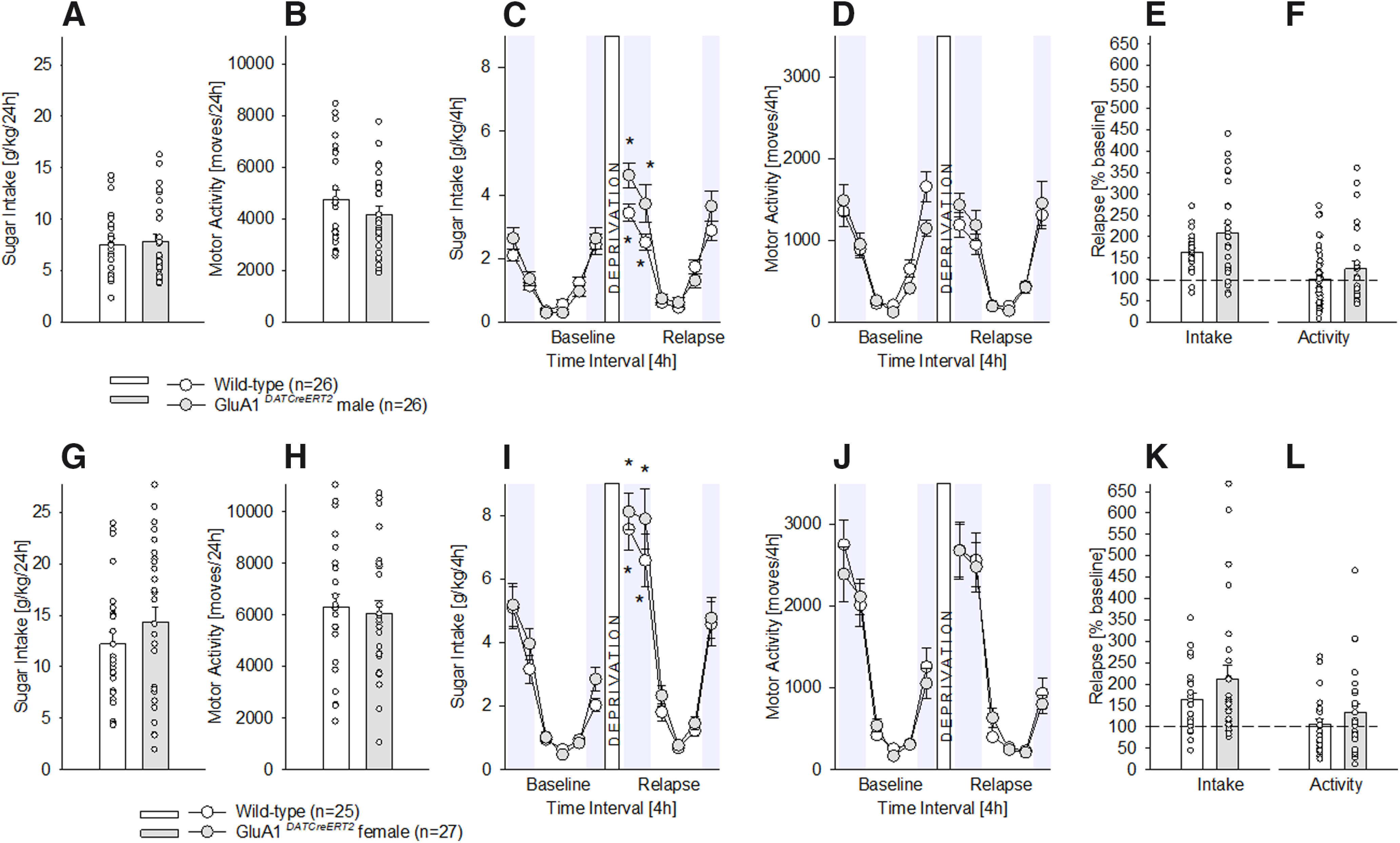
Characterization of chronic sugar drinking, SDE, and locomotor activity in male and female *GluA1^DATCreERT2^* mice. ***A***, ***G***, Baseline sugar intake in g/kg/d after free-choice access to a bottle containing a sugar solution (5% w/v) solution and a bottle with tap water in the homecage for eight weeks in wild-type male (*n* = 26), female (*n* = 25), and *GluA1^DATCreERT2^* male (*n* = 26) and female (*n* = 27) mice. ***B***, ***H***, Baseline locomotor activity after eight weeks of free-choice sugar drinking. ***C***, ***I***, Diurnal drinking pattern during baseline sucrose consumption and during the SDE. ***D***, ***J***, Diurnal activity pattern during baseline sucrose consumption and during the SDE. ***E***, ***K***, Relapse behavior measured in % change from baseline during the first 4 h of the SDE. ***F***, ***L***, Relative change in locomotor activity during the first 4 h of the SDE in relation to baseline activity; * indicates significant changes (*p* < 0.01) in sucrose consumption or activity during the SDE when compared with baseline.

Deletion of the AMPA receptor GluA1 subunit onto DAT-containing neurons did not have any effect on chronic sugar drinking, as the phenotypes displayed by both male (Fig. 3*A*,*B*) and female (Fig. 3*G*,*H*) *GluA1^DATCreERT2^* mice did not differ from their respective wild-type littermates after eight weeks of chronic, 24-h free-choice sugar exposure. As shown in Figure 3, daily sugar consumption (one-way ANOVA for Fig. 3*A*: *F*_(1,50)_ = 0.1, *p *=* *0.7 and for Fig. 3*G*: *F*_(1,50)_ = 1.3, *p *=* *0.3) as well as locomotor activity (Fig. 3*B*: *F*_(1,50)_ = 1.5, *p *=* *0.2 and Fig. 3*H*: *F*_(1,50)_ = 0.1, *p *=* *0.7) remained unaltered in the mutants.

Deletion of the AMPA receptor GluA1 subunit onto DAT-containing neurons did not have a role in relapse to sugar, as measured by the SDE (Fig. 3*C–F*, *I–L*). During baseline and SDE, the intake during 4-h intervals resembled the one already observed with *GluN1* mutations. That is, all mice showed the typical diurnal pattern of consumption, and a strongly pronounced sugar intake during the first 4–8 h of re-exposure which lasted not longer than 24 h. Statistical analysis showed a deprivation effect for male (Fig. 3*C*: *F*_(11,550)_ = 60.7, *p *<* *0.0001, Neuman–Keuls *post hoc p* < 0.01) and female (Fig. 3*I*: *F*_(11,550)_ = 85.2, *p *<* *0.0001, Neuman–Keuls *post hoc p* < 0.01), which was not GluA1 dependent (gene effect for Fig. 3*C*: *F*_(1,50)_ = 2.4, *p *=* *0.1 and for Fig. 3*I*: *F*_(1,50)_ = 1, *p *=* *0.3). Locomotor activity did not differ between baseline and SDE (Fig. 3*D*,*J*) for mutants and controls (two-way ANOVA for Fig. 3*D*: *F*_(1,50)_ = 0.1, *p *=* *0.7 and for Fig. 3*J*: *F*_(1,50)_ = 0.1, *p *=* *0.8), although statistical analysis showed a deprivation effect (Fig. 3*D*: *F*_(11,550)_ = 42, *p *<* *0.001 and Fig. 3*J*: *F*_(11,550)_ = 70, *p *<* *0.001). In line with these results, the percentage of relapse over baseline during the first 4 h of sugar re-exposure for intake and locomotion, did not differ in male (Fig. 3*E*: *F*_(1,50)_ = 3.9, *p *=* *0.06 and Fig. 3*F*: *F*_(1,50)_ = 1.1, *p *=* *0.3) or female (Fig. 3*K*: *F*_(1,50)_ = 0.7, *p *=* *0.4 and Fig. 3*L*: *F*_(1,50)_ = 0.7, *p *=* *0.4) *GluA1^DATCreERT2^*mice from their wild-type littermates.

**Figure 4.**
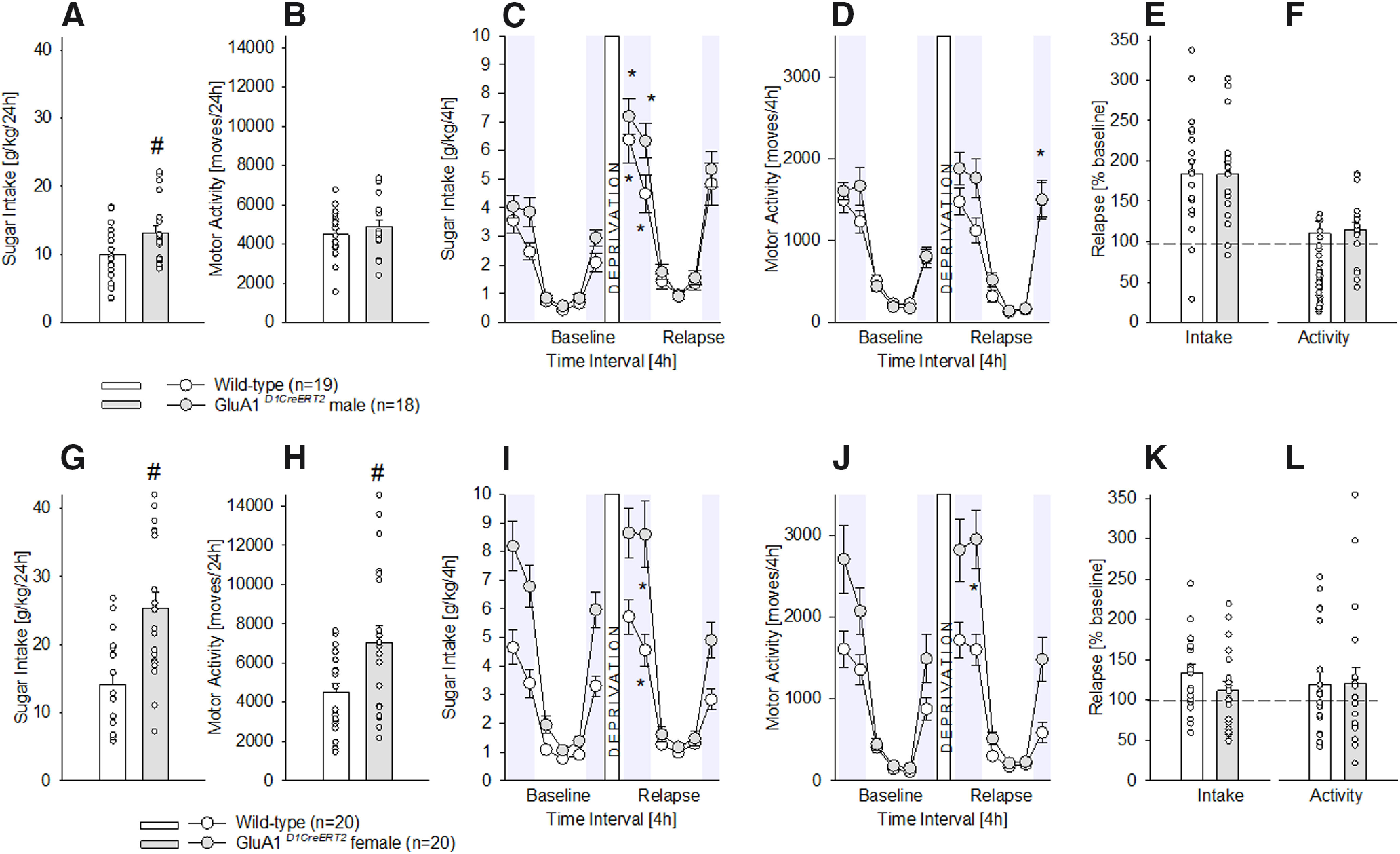
Characterization of chronic sugar drinking, SDE, and locomotor activity in male and female *GluA1^D1CreERT2^* mice. ***A***, ***G***, Baseline sugar intake in g/kg/d after free-choice access to a bottle containing a sugar solution (5% w/v) solution and a bottle with tap water in the homecage for eight weeks in wild-type male (*n* = 19), female (*n* = 20), and *GluA1^D1CreERT2^* male (*n* = 18) and female (*n* = 20) mice. ***B***, ***H***, Baseline locomotor activity after eight weeks of free-choice sugar drinking. ***C***, ***I***, Diurnal drinking pattern during baseline sucrose consumption and during the SDE. ***D***, ***J***, Diurnal activity pattern during baseline sucrose consumption and during the SDE. ***E***, ***K***, Relapse behavior measured in % change from baseline during the first 4 h of the SDE. ***F***, ***L***, Relative change in locomotor activity during the first 4 h of the SDE in relation to baseline activity; * indicates significant changes (*p* < 0.05) in sucrose consumption or activity during the SDE when compared with baseline; # indicates significant (*p* < 0.05) genotype differences.

As observed with the *GluN1* mutant mice, *GluA1* mutation onto D1-containing neurons (in contrast to the DAT-containing neurons), had an effect on chronic sugar drinking behavior (Fig. 4). That is, regardless of the sex, *GluA1^D1CreERT2^*mice showed a significant increase in the total, 24-h free-choice sugar drinking (one-way ANOVA, Fig. 4*A*: *F*_(1,35)_ = 4.3, *p *<* *0.05 and Fig. 4*G*: *F*_(1,38)_ = 17.1, *p *<* *0.001). Regarding locomotor activity, it did not change in male mutants (Fig. 4*B*: *F*_(1,35)_ = 0.9, *p *=* *0.3), but it was significantly increased in female *GluA1^D1CreERT2^*mice (Fig. 4*H*: *F*_(1,38)_ = 6.9, *p *<* *0.05).

Similar to the DAT-containing mutant mice, *GluA1* mutation onto D1-containing neurons did not influence the SDE (Fig. 4), and all mice showed increased sugar intake after deprivation during the first 4–8 h of re-exposure (two-way ANOVA, deprivation effect for Fig. 4*C*: *F*_(11,550)_ = 60.7, *p *<* *0.001 and Fig. 4*I*: *F*_(11,418)_ = 73, *p *<* *0.001, Neuman–Keuls *post hoc p* < 0.05). However, locomotor activity was not only influenced by a sugar deprivation period (Fig. 4*D*: *F*_(11,550)_ = 55, *p *<* *0.001 and Fig. 4*J*: *F*_(11,418)_ = 55.6, *p *<* *0.001, Neuman–Keuls *post hoc p* < 0.05), but also by the GluA1 receptor (two-way ANOVA, gene effect: Fig. 4*D*: *F*_(1,50)_ = 4.2, *p *<* *0.05 and Fig. 4*J*: *F*_(1,38)_ = 12.3, *p *<* *0.01; gene × deprivation interaction effect: Fig. 4*D*: *F*_(11,550)_ = 1.8, *p *<* *0.001 and Fig. 4*J*: *F*_(11,418)_ = 4.6, *p *<* *0.001) as only the male and female mutants showed an increased locomotor activity at the end of the SDE period. Calculating the percentage of relapse during the first 4 h of sugar re-exposure for intake and locomotion similar SDE magnitudes were found in both males (Fig. 4*E*: *F*_(1,50)_ = 0.006, *p *=* *1 and Fig. 4*F*: *F*_(1,50)_ = 0.06, *p *=* *0.08) and females (Fig. 4*K*: *F*_(1,38)_ = 2, *p *=* *0.2 and Fig. 4*L*: *F*_(1,38)_ = 0.005, *p *=* *0.9) compared with their respective wild-type littermates.

## Discussion

Here, we report on three findings in different inducible mouse mutant lines lacking either GluN1 or GluA1 receptor subunits in DAT or D1-containing neurons in a chronic free-choice sugar consumption paradigm and the SDE model. First, long-term sugar intake is modulated by AMPA and NMDA receptors in D1-containing neurons in an opposing manner. The specific deletion of the GluA1 subunit, which yields non-functional AMPA receptors in primarily D1-containing MSNs, increases excessive sugar drinking in male and female mice, whereas mice with inducible GluN1 receptor subunit deletion in D1-expressing neurons show significantly reduced chronic sugar intake. Second, neither AMPA nor NMDA receptors in DA neurons influence the development and maintenance of sugar consumption. Third, female *GluN1^D1CreERT2^* mice show a more pronounced relapse-like behavior in the SDE model.

The four genetic mouse models used here have some advantages over other approaches for gene targeting, allowing a more precise demonstration of the functional role regarding the gene of interest. First, these mutant models have high specificity of the deletion of GluN1 or GluA1 in DAT-expressing or D1-expressing neurons, as shown by previous co-localization studies (Engblom et al., 2008; Eisenhardt et al., 2015a). Cre-expression patterns fit with that described for DAT with strong expression in the VTA and for D1Rs with strong expression in the NAc and dorsal striatum. From previous studies (Engblom et al., 2008; Eisenhardt et al., 2015a), we conclude that we primarily have an ablation of individual glutamate receptor subunits within the mesolimbic DA system in our four mutant mouse lines. Second, the use of a temporally controlled gene deletion (induced by tamoxifen injections) circumvents potential developmental compensatory mechanisms, which may offset the loss of the gene and consequently mask its functional role.

As previously reported (Wei et al., 2021), mice show a typical diurnal pattern of sugar consumption. In all four mouse lines, such a pattern is maintained, and females showed consistently higher intake of a sugar solution relative to male mice. Long-term sugar intake was significantly more pronounced in *GluA1^D1CreERT2^* male and female mice, suggesting that functional AMPA receptors onto D1-containing neurons play a role in the regulation of excessive sugar consumption. Our finding largely agrees with a previous report that studied the regulation of AMPA receptors on NAc synapses by sucrose intake. Tukey et al. (2013) showed that repeated daily ingestion of a sucrose solution potentiated accumbal synapses through incorporation of calcium permeable AMPA receptors. In contrast, deletion of functional NMDA receptors on D1-expressing neurons reduced excessive sugar intake in both sexes. This finding is consistent with the few studies to date that have addressed the role of NMDA receptors in food or sugar binging, which have reported a reduction after systemic administration of the NMDA receptor antagonist memantine (Bisaga et al., 2008; Popik et al., 2011). A reduction of binge eating following memantine treatment was also seen in a human study (Brennan et al., 2008). These results and the fact that our *GluN1^DATCreERT2^* and *GluN1^D1CreERT2^* mutant mice showed no change in sugar binging suggest that non-mesolimbic brain regions may also contribute to sugar binging.

In terms of relapse-like behavior, we tested the four mutant mouse lines in the SDE model and found that neither AMPA nor NMDA receptors in DA neurons influenced augmented sugar consumption following a deprivation period. However, female *GluN1^D1CreERT2^* mutant mice showed a more pronounced SDE. In contrast, relapse behavior to drugs of abuse is strongly under the control of mesolimbic glutamate receptors, especially the ADE, like the SDE a measure of relapse-like behavior (Vengeliene et al., 2014). All four mouse mutant lines tested here were also tested in a previous study for alcohol relapse behavior. All mutant mice showed a significantly reduced ADE, results supported by intra-VTA and intra-accumbal pharmacological blockade of AMPA and NMDA receptors (Eisenhardt et al., 2015a). Similarly, the AMPA antagonist GYKI 54 266 completely abolished the ADE in rats (Sanchis-Segura et al., 2006) and a variety of NMDA receptor antagonists dose-dependently inhibited the ADE in rats (Hölter et al., 1996, 2000; Vengeliene et al., 2005; Kolik et al., 2017). For other drugs of abuse there are also consistent findings demonstrating that mesolimbic AMPA and NMDA receptors are critical for relapse behavior. Accumbal blockade of AMPA or NMDA receptors by various antagonists blocks relapse behavior for cocaine, heroin, and nicotine (Bäckström and Hyytiä, 2007; LaLumiere and Kalivas, 2008; Gipson et al., 2013; Doyle et al., 2014) and deletion of NMDA receptors in D1-containing neurons reduces the incubation of cocaine-seeking and relapse (Mameli et al., 2009). In conclusion, pharmacological inhibition or genetic inactivation of acccumbal NMDA receptors reduces relapse to drugs of abuse/alcohol, whereas *GluN1^D1CreERT2^* female mutant mice exhibit an augmented SDE.

In summary, a previous study demonstrated the occurrence of an addictive-like phenotype for sugar in male and female mice similar to that of drugs of abuse (Wei et al., 2021). Here, we show that mesolimbic AMPA and NMDA receptor do not play a critical role in relapse to sugar consumption. These findings differentiate a natural reward from drugs of abuse on the molecular level, as mesolimbic NMDA and AMPA receptors are essential for drug-induced neuroplasticity and subsequent relapse behavior.

## Synthesis

Reviewing Editor: Leandro Vendruscolo, The Scripps Research Institute Decisions are customarily a result of the Reviewing Editor and the peer reviewers coming together and discussing their recommendations until a consensus is reached. When revisions are invited, a fact-based synthesis statement explaining their decision and outlining what is needed to prepare a revision will be listed below. The following reviewer(s) agreed to reveal their identity: Giordano de Guglielmo.

I agree with the reviewers that:

1. Any statement of addictive-like behavior (Abstract), sugar-addicted (Significance statement), compulsive overconsumption, excessive (what is the operational definition of excessive here?), relapse, addictive properties of sugar, etc, should be deleted. For the previous literature, the authors should explicitly describe what has actually been measured. For the present study, description should be limited to sugar-deprivation effect.

Additionally, the authors should focus on the effects of chronic sugar consumption on reward systems instead of comparing with drugs of abuse.

2. Re-organize the Results section to describe all of the data showed in Figure 1 first, then follow with description of figures 2, 3, and 4.

3. The authors used tamoxifen (1 mg) to induce mutation. This information is critical for data interpretation, yet it is buried in Methods. It is unclear when tamoxifen was administered. Perhaps a timeline of the experiment should be added to indicate when tamoxifen was administered during the experiments.

4. The sample size should be indicated in the figure legends too. In the figure legends, avoid explanation about the SDE and references; this should be in Methods.

5. In Discussion, the wording about the procedure should be careful: “mice lacking NMDA receptors” versus “gene inactivation” versus “temporally controlled gene deletion.”

## Author Response

Point-by-point reply to the reviews for eNeuro eN-NWR-0029-21 “NMDA receptors in accumbal D1 neurons influence chronic sugar consumption and relapse"

Synthesis Statement from the Reviewing Editor:

1. Any statement of addictive-like behavior (Abstract), sugar-addicted (Significance statement), compulsive overconsumption, excessive, relapse, addictive properties of sugar, etc, should be deleted. For the previous literature, the authors should explicitly describe what has actually been measured. For the present study, description should be limited to sugar-deprivation effect.

Answer: For the eNeuro manuscript we have now focused on the sucrose-deprivation effect and have avoided terms such as sugar addiction etc. However, it is important to know that the manuscript we are referring to in the Introduction and Discussion (“Female mice are more prone to develop an addictive-like phenotype for sugar consumption” by Wei et al.) has now been accepted for publication in Scientific Reports. For that reason, we believe that discussing some of our findings from an addiction perspective is now justified. Nevertheless, following your suggestions, we have changed as much as possible the addiction relevant terminology.

Additionally, the authors should focus on the effects of chronic sugar consumption on reward systems instead of comparing with drugs of abuse.

Answer: The current manuscript aimed to specifically test the involvement of NMDA and AMPA receptors within the mesolimbic system in the SDE. Nevertheless, in the Introduction and Discussion we have mentioned the ADE as well.

2. Re-organize the Results section to describe all of the data showed in Figure 1 first, then follow with descriptions of figures 2, 3, and 4.

Answer: According to the reviewer’s comment, we have reorganized the structure of the results section (see detailed response to reviewer 2).

3. The authors used tamoxifen (1 mg) to induce mutation. This information is critical for data interpretation. It is unclear when tamoxifen was administered. Perhaps a timeline of the experiment should be added to indicate when tamoxifen was administered during the experiments.

Answer: We have added the following information in the animal section of materials and methods: “Mice were treated with tamoxifen at an age of 8-10 weeks old and were allowed to recuperate for at least 3 weeks before experiments started.”

4. The sample size should be indicated in the figure legends too. In the figure legends, avoid explanation about the SDE and references; this should be described in Methods.

Answer: The sample size is now indicated in the figure legends, and the sentence about SDE and references has been moved to the method section.

5. In Discussion, the wording about the procedure should be careful: “mice lacking NMDA receptors” versus “gene inactivation” versus “temporally controlled gene deletion.”

Answer: We have used these terms now more precisely.

Reviewer 1

This study evaluates the involvement of AMPA and NMDA receptors within mesolimbic circuitry on (presumably) sucrose consumption in male and female mice, using the inducible transgenic mutant lines GluA1D1CreERT2, GluN1D1CreERT2, GluA1DATCreERT2, and GluN1DATCreERT2. An examination of the neurobiology involved in addictive-like sucrose consumption behavior is certainly interesting and valuable. The premise, statistical analysis, and interpretation of the data would be acceptable.

However, it is not apparent based on the data presented in the manuscript that the sucrose consumption paradigm used can facilitate addictive-like sucrose consummatory behavior. The paper that is cited in reference to the model of addictive-like sucrose consumption is actually a preprint on bioRxiv. While I support the concept of pre-print publications and referencing them in manuscripts generally, I do not think it is appropriate to cite and additionally publish a scientific study in which the animal model itself has not been peer-reviewed and published. Upon reading that particular manuscript in which various sucrose? drinking pardigms were evaluated, it is still not clear how the model used in the present manuscript could be considered a model of addictive like sucrose consumption. Therefore I am unable to consider this manuscript acceptable for publication at this time.

Answer: We understand the reviewer’s point. At the time we submitted the present study, the manuscript that we cite was under revision. Now that manuscript has been accepted in Scientific Reports (see picture below). In that manuscript, like in the current study, we have tested long-term free choice home cage drinking procedure measuring the sugar deprivation effect (SDE) following an abstinence phase and have demonstrated the occurrence of an addictive-like phenotype for sugar in male and female mice, similar to that observed with other drugs of abuse such as alcohol.

Minor comments:

1. What sugar is being used in these experiments? Sucrose? This should be specified.

Answer: We have now specified in the material and methods section (“Home cage two-bottle free choice sugar drinking and assessment of relapse-like drinking by means of the sugar deprivation effect (SDE)” subsection, second sentence) that we used sucrose (5% w/v).

2. It is not clear from the methods section when baseline measurements of sugar and water intake were recorded, although the authors do state that sugar and water intake were recorded in the last 3 days of the 8 week sugar exposure. It would be helpful to specifically state this was the baseline measurement if that were the case.

Answer: We apologize for not being clear enough. The specific information has been added: “During the last 3 days of sugar exposure, sugar and water intake and locomotion were recorded using a Drinkometer system (Eisenhardt et al., 2015a, 2015b; Bilbao et al., 2019) and were used as baseline for comparison with the SDE.”

We thank the reviewer for her/his comments and we hope that the current version of the manuscript will be considered for publication.

Reviewer 2

The manuscript titled “NMDA receptors in accumbal D1 neurons influence chronic sugar consumption and relapse” investigate the contribution of mesolimbic glutamate receptors in mediating chronic sugar consumption and the sugar deprivation effect (SDE). The authors used four inducible mutant mouse lines lacking the GluA1 or GluN1subunit in either dopamine transporter (DAT) or D1R-expressing neurons and found altered sugar consumption in the mice lacking either GluA1 or GluN1 in D1R expressing neurons and a more pronounced SDE in the female GluN1D1CreERT2 mice. The main issues with this manuscript are the overall presentation of the findings and the statistical analysis:

The manuscript would benefit major changes in the description of the results. I recommend the authors describing the results of each figure separately, so the reader will not have to bounce between figures while reading the result section. Even if this could increase the length of the manuscript, it would make easier for the reader to follow and understand the results.

Answer: We thank the reviewer for this comment. We have changed the structure of the results section which allows now to describe each figure separately.

The statistical analysis is problematic and confusing:

1. Sex should be included as a factor in the ANOVAs throughout the paper. This is particularly important since the authors claim sex differences in the results.

Answer: We understand the point of the reviewer. We initially also considered the possibility of doing a sex comparison in addition to the factor gene. However, since we already compared sugar-related phenotypes in male and female mice in our recently accepted manuscript “Female mice are more prone to develop an addictive-like phenotype for sugar consumption", Scientific Reports) we think that including sex as an additional factor (on all levels of our analyses) will provide a too complex picture. Thus, discussing potential sex differences would only diffuse and confound the main question of the manuscript (does the NMDAR or AMPAR play a role in SDE?). Please note: sex differences are already observed in wild-type mice (see also Wei et al., 2021), suggesting hormonal or motivationally- driven mechanisms.

2. Newman Keuls post hoc test results are never described in the result section.

Answer: The reviewer is right. The post hoc values have been added in the results section.

3. For the SDE results a description of the analysis is missing. Which are the factors included in the analysis? It is not clear what was used as baseline for the comparison.

Answer: A specific description of the SDE analysis is now provided in the statistics section: “The ANOVA model for the long-term free choice home cage drinking and sugar deprivation effect (SDE) contained the fixed effects of sugar deprivation (baseline and relapse), gene (wild-type and GluA1 or GluN1) and the interaction deprivation x gene.” In addition, we have specified in the material and methods section (“Home cage two-bottle free choice sugar drinking and assessment of relapse-like drinking by means of the sugar deprivation effect (SDE)” subsection) what was used as baseline: “During the last 3 days of sugar exposure, sugar and water intake and locomotion were recorded using a Drinkometer system (Eisenhardt et al., 2015a, 2015b; Bilbao et al., 2019) and were used as baseline for comparison with the SDE.”

4. Asterisks in the figures indicate individual comparisons that are not justified given the lack of significant interaction in the ANOVA results (For example fig 1C,D,I)

Answer: The asterisks in the figures indicate post-hoc values whenever there was a deprivation or gene effects, or an interaction effect. Since we found at least a deprivation effect in all figures, we think is correct to add post-hoc values indicating differences between baseline and SDE.

Could the differences observed in sugar consumption be due to the sex differences in locomotor activities? For example, it seems that there is a big difference in locomotor activity between male and female mice in Fig. 1B, 3B.

Answer: The reviewer raises a very interesting point. We don’t think that the sex differences in sugar are related to locomotor activation. First, a higher locomotor activity (displayed by the females) could eventually lead to a lower consumption (the locomotion competes with drinking behavior). Second, although the higher locomotion observed in females a phenomenon commonly observed and relatively constant (regardless of sugar exposure), the sugar consumption is not always increased, as it is more context-dependent.

In page 11 there is a reference to a Fig 7, but there are only 4 figures in the manuscript

Answer: We apologize for this mistake, and this typo error has been corrected.

We thank the reviewers and the editorial board members for their comments and we hope that the current version of the manuscript will be considered for publication.
